# Comparison of Standard Automated Perimetry, Short-Wavelength Automated Perimetry, and Frequency-Doubling Technology Perimetry to Monitor Glaucoma Progression

**DOI:** 10.1097/MD.0000000000002618

**Published:** 2016-02-18

**Authors:** Rongrong Hu, Chenkun Wang, Yangshun Gu, Lyne Racette

**Affiliations:** From the Department of Ophthalmology, First Affiliated Hospital, College of Medicine, Zhejiang University, Hangzhou, China (RH, YG); Eugene and Marilyn Glick Eye Institute, Indiana University (RH, LR); and Indiana University, Fairbanks School of Public Health, IN, USA (CW).

## Abstract

Detection of progression is paramount to the clinical management of glaucoma. Our goal is to compare the performance of standard automated perimetry (SAP), short-wavelength automated perimetry (SWAP), and frequency-doubling technology (FDT) perimetry in monitoring glaucoma progression.

Longitudinal data of paired SAP, SWAP, and FDT from 113 eyes with primary open-angle glaucoma enrolled in the Diagnostic Innovations in Glaucoma Study or the African Descent and Glaucoma Evaluation Study were included. Data from all tests were expressed in comparable units by converting the sensitivity from decibels to unitless contrast sensitivity and by expressing sensitivity values in percent of mean normal based on an independent dataset of 207 healthy eyes with aging deterioration taken into consideration. Pointwise linear regression analysis was performed and 3 criteria (conservative, moderate, and liberal) were used to define progression and improvement. Global mean sensitivity (MS) was fitted with linear mixed models.

No statistically significant difference in the proportion of progressing and improving eyes was observed across tests using the conservative criterion. Fewer eyes showed improvement on SAP compared to SWAP and FDT using the moderate criterion; and FDT detected less progressing eyes than SAP and SWAP using the liberal criterion. The agreement between these test types was poor. The linear mixed model showed a progressing trend of global MS overtime for SAP and SWAP, but not for FDT. The baseline estimate of SWAP MS was significantly lower than SAP MS by 21.59% of mean normal. FDT showed comparable estimation of baseline MS with SAP.

SWAP and FDT do not appear to have significant benefits over SAP in monitoring glaucoma progression. SAP, SWAP, and FDT may, however, detect progression in different glaucoma eyes.

## INTRODUCTION

The detection of progression is one of the most important and challenging aspects in the clinical management of glaucoma. Although both structural and functional changes can occur overtime, functional progression correlates more closely with quality of life for glaucoma patients.^1,2^ White-on-white standard automated perimetry (SAP) remains the reference standard to detect glaucomatous visual field loss. Alternative perimetric test types, however, have been developed based on the hypothesis that reducing the redundancy within the visual system may facilitate the detection of visual field loss. Short-wavelength automated perimetry (SWAP) and frequency-doubling technology perimetry (FDT) are 2 types of perimetry that have received wide interest. SWAP targets the koniocellular pathway^3^ and FDT targets the magnocellular pathway,^4^ though recent studies show that other types of retinal ganglion cells and cortical factors may also mediate the detection of the FDT stimulus.^5–7^ Although a considerable number of studies have shown that SWAP^8–12^ and FDT^10,12–16^ results can predict the future onset of visual field loss with SAP, other studies have questioned the advantage of these tests over SAP.^17–20^

The ability of SWAP and FDT to monitor glaucomatous progression in patients with established open-angle glaucoma remains unclear. Evidence derived from the 1st generation of these tests has suggested the possibility of better performance in detecting progression compared to SAP.^21–24^ In contrast to the full-threshold SWAP, the Swedish Interactive Thresholding Algorithm (SITA) SWAP has shortened test duration and reduced measurement variability.^25^ The 2nd generation of FDT, the Matrix, increases the spatial resolution by using a 24-2 testing pattern similar to SAP. Unlike for SAP, measurement variability does not increase with the deterioration of sensitivity for either generation of FDT.^26–28^ A small-sample experimental study also showed less intra- and intertest variability with FDT compared to SAP by analyzing the frequency-of-seeing curves.^29^ Although these properties may provide potential advantages for FDT in monitoring glaucomatous progression, the results from several recent longitudinal studies do not conclusively show an advantage for FDT compared to SAP.^16,30–33^

A direct comparison of the results of different perimetric tests is complicated by several challenges, including the use of different stimuli and measurement scales. Each test uses a different type of stimulus that is defined by different types of contrast. SAP uses a white stimulus presented on a white background, which can be defined by Weber contrast. SWAP uses a blue stimulus presented on a bright yellow background, which can also be defined by Weber contrast. The stimulus used in FDT consists of a sinusoidal grating of low spatial frequency that undergoes counterphase flickering at a high temporal frequency, and can be defined by Michelson contrast. Although the sensitivity values of these 3 test types are expressed in decibels (dB), their measurement scales differ conceptually and have different dynamic ranges and intervals. As a result, a 1 dB sensitivity loss per year on SAP cannot be assumed to be equivalent to a 1 dB sensitivity loss per year on FDT. These challenges can be overcome by converting the data into a common and comparable scale by expressing the sensitivities as contrast sensitivity^34^ and in percent of mean normal.^35–37^ The goal of the present study is to compare SAP, SWAP, and FDT in their ability to detect progression once all data are expressed in a comparable scale.

## METHODS

### Participants

All participants were selected from the Diagnostic Innovations in Glaucoma Study (DIGS) and the African Descent and Glaucoma Evaluation Study (ADAGES), which have been described in detail elsewhere.^38^ In brief, these longitudinal studies are prospectively designed to assess structure and function in glaucoma. These multicenter studies were approved by all appropriate Institutional Review Boards, adhered to the tenets of the declaration of Helsinki for research involving human subjects, and were performed in conformity with the Health Insurance Portability and Accountability Act.

Participants underwent comprehensive ophthalmic examinations, including review of medical history, best-corrected visual acuity, slit-lamp biomicroscopy, intraocular pressure (IOP) measurement, gonioscopy, dilated funduscopic examination, and stereoscopic optic disk photography. All participants had open angles, best-corrected acuity of 20/40 or better, spherical refraction within 5.0 diopters, and cylinder correction within 3.0 diopters. Participants were excluded if they had a history of intraocular surgery (except for uncomplicated cataract surgery); secondary causes of elevated IOP (eg, iridocyclitis, trauma); other systemic or ocular diseases known to affect the visual field (eg, pituitary lesions, demyelinating diseases, human immunodeficiency virus positive or acquired immune deficiency syndrome, or diabetes); medications known to affect visual field sensitivity; and an inability to perform visual field examinations reliably or life-threatening diseases.

### Inclusion Criteria for the Present Study

The present study included 113 eyes of 84 patients longitudinally followed with SAP, SWAP, and FDT. Of these, 98 eyes had documented glaucomatous optic neuropathy by stereophotographs and 15 eyes had documented ocular hypertension.^38^ To be included in this study, patients had to have at least 5 visits (range, 5–7 visits). At each visit, patients had a reliable SAP, SWAP, and FDT test taken within a 30-day window. A minimum of 3 months separated each of the consecutive visits. At the baseline of the present study (visit 1), all eyes had or had a history of having at least 1 abnormal SAP, 1 abnormal SWAP, and 1 abnormal FDT (abnormality defined in the “Visual Field Tests” section). The visual field abnormality was confirmed on at least one of these test types at study baseline.

### Visual Field Tests: SAP, SWAP, and FDT

We included SAP-SITA and SWAP-SITA tests taken with the 24-2 pattern on the Humphrey Field Analyzer (Carl Zeiss Meditec, Dublin, CA). The FDT results were taken with the 24-2 pattern and Zippy Estimation by Sequential Testing thresholding algorithm on the Humphrey Matrix FDT Perimeter (Carl Zeiss Meditec Inc., Dublin, CA) using Welch-Allyn technology. All visual fields were evaluated by the Visual Field Assessment Center at the Department of Ophthalmology, University of California, San Diego.^39^ Only reliable visual fields, defined as ≤33% fixation losses, false-negative responses, and false-positive responses, were included. Visual fields with artifacts (eg, lid and lens rim artifacts) were excluded.

Visual field results were considered abnormal if one of the following criteria was met: the pattern standard deviation (PSD) was triggered at *P* < 5% or worse level; the Glaucoma Hemifield Test result was “outside normal limits”; or the presence of a cluster of 3 or more nonedge points, all of which triggered at *P* < 5% level with at least 1 triggered at *P* < 1% level in the pattern deviation plot.^40^ The same criteria were applied for SAP, SWAP, and FDT.

### Conversion of Units for SAP, SWAP, and FDT

In order to have a common scale across all test types, we 1st converted the sensitivity values from dB to linear contrast sensitivity at each test location using the approach outlined by Sun et al.^34^ Contrast sensitivity is a unitless measure, which is the reciprocal of contrast threshold. For SAP and SWAP, Weber contrast is used, which is the luminance increment divided by the mean luminance; for FDT, this is equivalent to Michelson contrast.^34,41^ Then we further expressed the values as percent of mean normal by dividing them by the normal sensitivity of that age at each location, which was also converted as linear contrast sensitivity. Percent of mean normal is a relative scale that provides an intuitive estimate of glaucomatous status regardless of the type of measurements and has been used in previous studies.^35–37^

The normal sensitivity values were estimated from an independent cross-sectional dataset of 207 participants, which was also selected from the DIGS and ADAGES studies and covered the same age range with the patient dataset. These participants had healthy eyes, IOP < 22 mmHg (no history of ocular hypertension), and normal appearing optic discs by stereophotograph assessment.^38^ They had normal visual fields on SAP, SWAP, and FDT (or no confirmed abnormal visual field results). One eye of each participant was randomly selected for analysis. For each eye, SAP, SWAP, and FDT were taken within 30 days of each other. To take the deterioration of sensitivity due to aging into consideration, ordinary least squares linear regression of sensitivity (in dB) versus age (independent variable) was computed for each test location. Significant negative relationships were obtained between age and sensitivity for each test type, and the linear regression was used to compute the mean normal sensitivity as a function of age.

### Pointwise Linear Regression (PLR) Analyses

We performed PLR analyses to determine whether change (progression or improvement) occurred at each visual field location overtime.^42–44^ Although there is no “gold standard” for progression using PLR,^45,46^ the commonly applied criterion of slope with SAP is more than 1 dB sensitivity loss per year at a significant level;^32,43–45,47–51^ and as the edge locations are subject to more variability,^52^ a steeper slope of 2 dB loss per year has been adopted for them.^32,43,44,47^ Because we did not use the decibel scale in this study, we approximated what 1 and 2 dB loss per year would translate to in percent of mean normal. For example, consider a 50-year-old patient with 29 dB sensitivity at a given nonedge location and 27 dB sensitivity at a given edge location, respectively, progressing at 1 and 2 dB per year. After conversion, the baseline sensitivity would be 48.2% and 55.0% of mean normal, respectively. With PLR, the slopes of sensitivity overtime would correspond to 6.8% and 11.2% of mean normal loss per year, respectively. Hence, we approximated the cut-off criteria to be 5% of mean normal loss per year for nonedge locations and 10% of mean normal loss per year for edge locations for SAP, SWAP, and FDT. Test locations were therefore flagged as statistically significant progression if the slope of sensitivity overtime was ≤−5% of mean normal per year for nonedge locations, and ≤−10% of mean normal per year for edge locations, with *P* < 0.05. On the other hand, a test location would be flagged as improvement if the regression slope was ≥5% of mean normal per year for nonedge locations, and ≥10% of mean normal per year for edge locations, with *P* < 0.05.

To determine whether a given eye was changing (progressing or improving) overtime, 3 different criteria were used:

1. A conservative criterion in which at least 3 adjacent locations in the same hemifield were flagged as progression (Prog_Cons) or improvement (Imp_Cons) with at least one nonedge location
2. A moderate criterion in which any 3 locations within the visual field were flagged as progression (Prog_Mod) or improvement (Imp_Mod) with at least 1 nonedge location
3. A liberal criterion in which any 2 locations were flagged as progression (Prog_Lib) or improvement (Imp_Lib) with at least 1 nonedge location

The same criteria were applied to SAP, SWAP, and FDT. If a given eye met the criteria for both progression and improvement, we defined that eye as indeterminate with regard to the direction of change. The criteria of improvement were set to work as a proxy of specificity of PLR analysis.^30^

### Global and Sectoral Analyses

To calculate the global mean sensitivity (MS) in percent of mean normal, the sensitivity values of each location excluding the 2 above and below the blind spot were first converted to linear contrast sensitivity and then averaged to obtain the MS. The same treatment was applied to obtain the age-matched normal MS in linear contrast sensitivity. The final values in percent of mean normal for global MS were obtained by dividing patients’ MS in linear contrast sensitivity by the normal MS in linear contrast sensitivity. Analogously, the MS in percent of mean normal was separately calculated for the supero-temporal (ST) sector and infero-temporal (IT) sector.^53,54^ The rationale for converting from logarithmic to linear units before averaging has been outlined by Hood et al.^55^

### Statistical Analyses

The Bland–Altman analysis was used to assess the agreement between different test types in estimating baseline global MS (in percent of mean normal). The Cochran Q test was used to compare the proportion of progressing and improving eyes across all test types and if a significant difference was found, the McNemar test was used to determine which pairs of tests differed from each other. The Fleiss Kappa (κ) was used to evaluate the agreement of progression among different test types and the *P* value was approximated using the Monte Carlo test.^56^ A value <0.0 indicates poor agreement, and 0.01 to 0.20 as slight, 0.21 to 0.40 as fair, 0.41 to 0.60 as moderate, 0.61 to 0.80 as substantial, and 0.81 to 1.0 as almost perfect.^57^ The agreement of progression was further assessed with the interclass correlation coefficients (ICC), which were calculated using two-way random single measures. The Friedman test was used to compare the number of progressing and improving eyes at each test location across all 3 types and if a significant difference was found, the Wilcoxon signed-rank test was used to determine which pairs of tests differed from each other. *P* < 0.05 was considered statistically significant in all analyses.

Longitudinal MS data (global, ST and IT sectors) were fitted by linear mixed models. Follow-up time, test type, and their interaction were considered as the fixed effect. Random intercepts and slopes were included at the subject level. Random intercepts were included at eye levels with 2 eyes nested within each subject. Comparisons among the main effect of test types and the rates of change of MS among test types (interaction effect) were conducted by the Wald test. SAP was considered as the reference type. All analyses were carried out in R^58^ and SAS (version 9.4; SAS Institute, Inc., Cary, NC). The R package visualFields^59^ was used to process the visual field data.

## RESULTS

**FIGURE 1:**
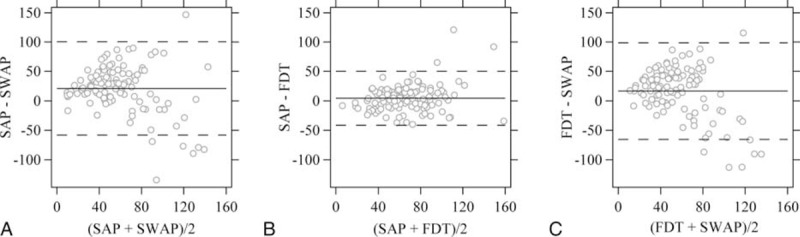
Limits of agreement between different test types on the estimate of global mean sensitivity in percent of mean normal. (A) Between SAP and SWAP; (B) between SAP and FDT; and (C) between SWAP and FDT. The horizontal axis shows the average global mean sensitivity with each pair of tests for each eye. The vertical axis shows the difference of global mean sensitivity within each pair of tests. The solid lines and the dashed lines represent the mean difference and corresponding 95% limits of agreement. FDT = frequency-doubling technology perimetry, SAP = standard automated perimetry, SWAP = short-wavelength automated perimetry.

At baseline, the mean age of the 84 glaucoma patients (113 eyes) included in this study was 60.2 with a standard deviation of 9.1 years. Fifty patients (59.5%) were female. The mean follow-up of visual field tests available for PLR analysis in each eye was 4.4 years (range, 3.1–5.5). The mean interval between follow-up visits in each eye was 12.0 months with a standard deviation of 3.3 months. Table 1 shows the median, and 1st and 3rd quartiles of MD and PSD for SAP, SWAP, and FDT tests at baseline. The global indices of the normal dataset are also shown in Table 1. As shown in Figure 1, SAP and FDT had better agreement in estimating baseline global MS in comparison with SWAP.

**TABLE 1:**
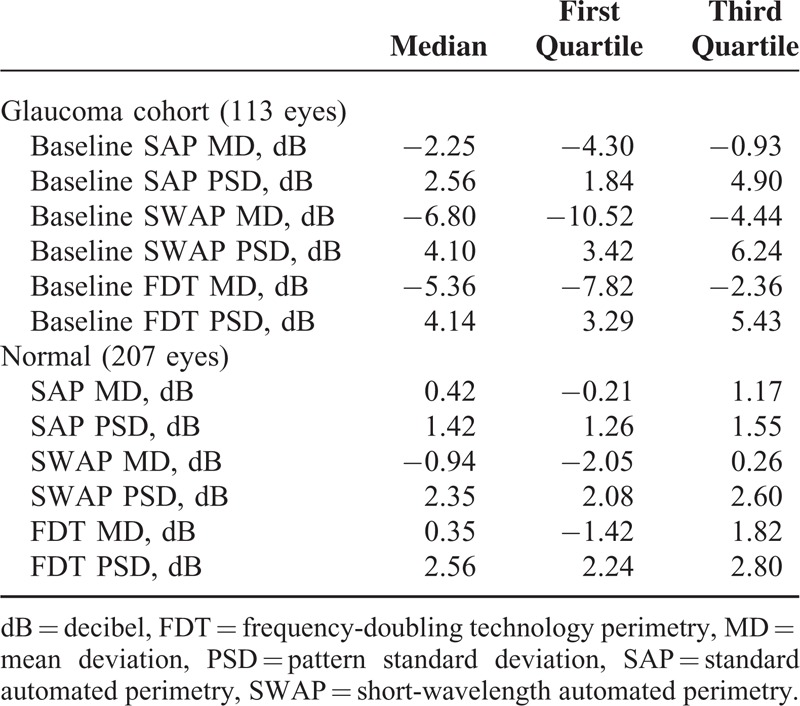
Global Indices of Visual Field Measurements

### Pointwise Linear Regression Analyses

**FIGURE 2:**
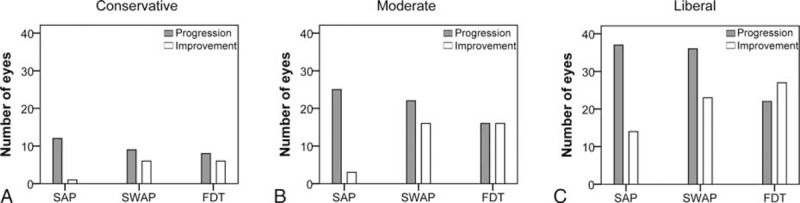
The number of eyes classified as progression (gray bar) or improvement (white bar) using different levels of criteria. (A) Conservative criteria Prog_Cons and Imp_Cons; (B) moderate criteria Prog_Mod and Imp_Mod; and (C) liberal criteria Prog_Lib and Imp_Lib.

Using the criterion Prog_Cons, there were 12 (10.6%), 9 (8.0%), and 8 (7.1%) eyes classified as progressing by SAP, SWAP, and FDT (Figure 2A). Using the criterion Prog_Mod, there were 25 (22.1%), 22 (19.5%), and 16 (14.2%) eyes classified as progressing, respectively (Figure 2B). Using the criterion Prog_Lib, there were 37 (32.7%), 36 (31.9%), and 22 (19.5%) eyes classified as progressing, respectively (Figure 2C). FDT classified significantly less progressing eyes than SAP and SWAP (*P* = 0.017) with the criterion Prog_Lib and no significant difference was observed with criteria Prog_Cons (*P* = 0.568) and Prog_Mod (*P* = 0.223).

Using the criterion Imp_Cons, there were 1 (0.9%), 6 (5.3%), and 6 (5.3%) eyes classified as improvement by SAP, SWAP, and FDT (Figure 2A). Using the criterion Imp_Mod, there were 3 (2.7%), 16 (14.2%), and 16 (14.2%) eyes classified as improvement, respectively (Figure 2B). Using the criterion Imp_Lib, there were 14 (12.4%), 23 (20.4%), and 27 (23.9%) eyes classified as improvement, respectively (Figure 2C). SAP classified significantly less improving eyes than SWAP and FDT (*P* = 0.004) with the criterion Imp_Mod and no significant difference was observed with criteria Imp_Cons (*P* = 0.125) and Imp_Lib (*P* = 0.056).

With the Prog_Cons criterion, no eyes were classified as indeterminate. One eye was classified as indeterminate by SAP using the Prog_Mod criterion (this eye was classified as progression by FDT), and 4 eyes were classified as indeterminate by SAP using the Prog_Lib criterion (2 of which were classified as progression by both SWAP and FDT, and 1 as progression by SWAP).

**FIGURE 3:**
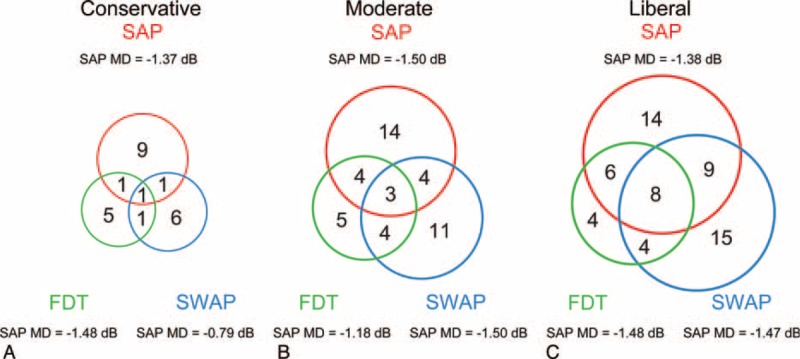
Venn diagrams showing the number of progressing eyes detected by SAP, SWAP, and FDT using different levels of criteria. (A) Conservative criterion Prog_Cons; (B) moderate criterion Prog_Mod; and (C) liberal criterion Prog_Lib. The medians of baseline MD in dB for progressing eyes detected by each test type are shown. For direct comparison, all the MD values are from SAP. dB = decibel, FDT = frequency-doubling technology perimetry, MD = mean deviation, SAP = standard automated perimetry, SWAP = short-wavelength automated perimetry.

The agreement of progression classification between test types was slight to fair and the κ was 0.13 (range, 0.03–0.24), 0.18 (range, 0.07–0.29), and 0.24 (range, 0.13–0.35), respectively, with the criteria Prog_Cons, Prog_Mod, and Prog_Lib (Figure 3). The ICC between test types was 0.14 (range, 0.02–0.26), 0.19 (range, 0.07–0.31), and 0.25 (range, 0.13–0.37), respectively, with the criteria Prog_Cons, Prog_Mod, and Prog_Lib.

**FIGURE 4:**
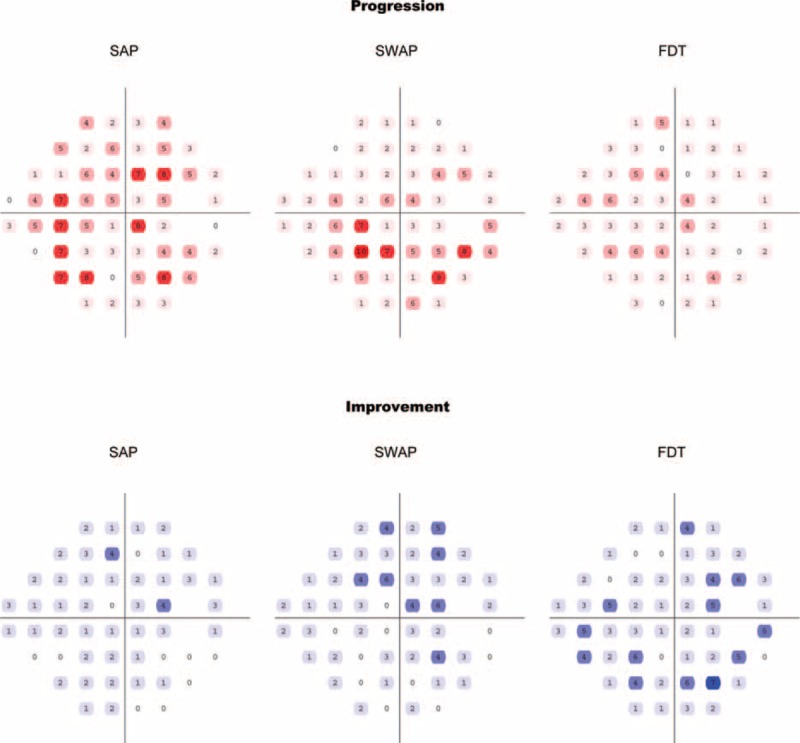
The number of progression (upper panel) and improvement (lower panel) eyes on each test location detected by SAP, SWAP, and FDT in the study cohort. Deep shades represent higher numbers. FDT = frequency-doubling technology perimetry, SAP = standard automated perimetry, SWAP = short-wavelength automated perimetry.

Figure 4 shows the number of eyes with progression and improvement at each test location detected by SAP, SWAP, and FDT. Taking all 52 locations (excluding the blind spots) into consideration, SAP detected more progression than SWAP and FDT (*P* = 0.008) and less improvement than SWAP and FDT (*P* = 0.018).

**FIGURE 5:**
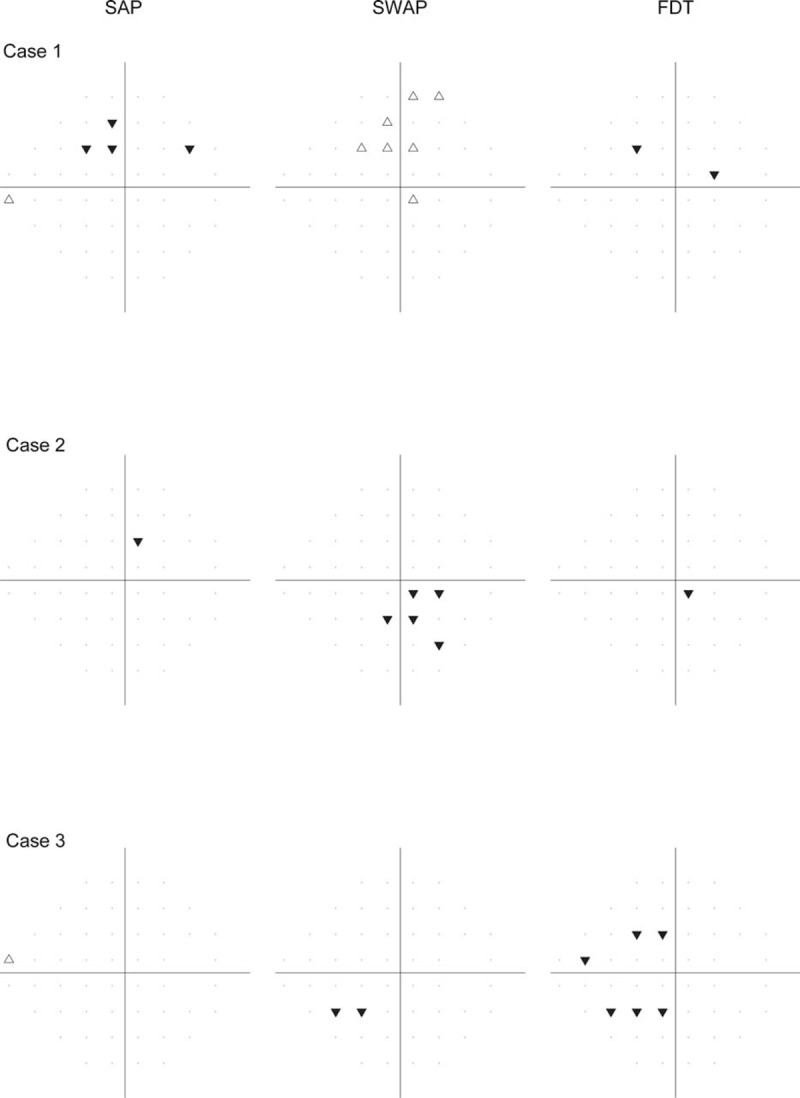
Three cases analyzed with the pointwise linear regression analyses. The inverted solid (▾) and regular empty triangles (Δ) represent the progression and improvement, respectively.

Three cases of PLR analysis are shown as examples in Figure 5. The agreement in the spatial location of progression with different test types was poor. For Case 1, SAP and SWAP flagged the same locations with opposite directions of change in the supero-nasal area, where SAP detected progression while SWAP reported improvement. For Case 2, SWAP detected a cluster of progressing locations in the infero-temporal area, while SAP and FDT did not detect such changes. For Case 3, SWAP and FDT had partial agreement for the progression in the infero-nasal area, while SAP did not detect progression within the visual field.

### Global and Sectoral Progression Analyses Using Linear Mixed Modeling

Table 2 presents the results from multilevel mixed effect model with global MS, ST MS, and IT MS, respectively, as dependent variables. With SAP, the global MS in percent of mean normal was described by a linear mixed model as, MS_SAP-global_ (time) = 71.10 − 1.69 ∗ time, where time refers to the follow-up duration in year and was set at 0 for baseline. The rate of change of global MS, −1.69% of mean normal per year was significantly different from zero (*P* = 0.011, 95% confidence limit [CL], −2.97 to −0.41).

**TABLE 2:**
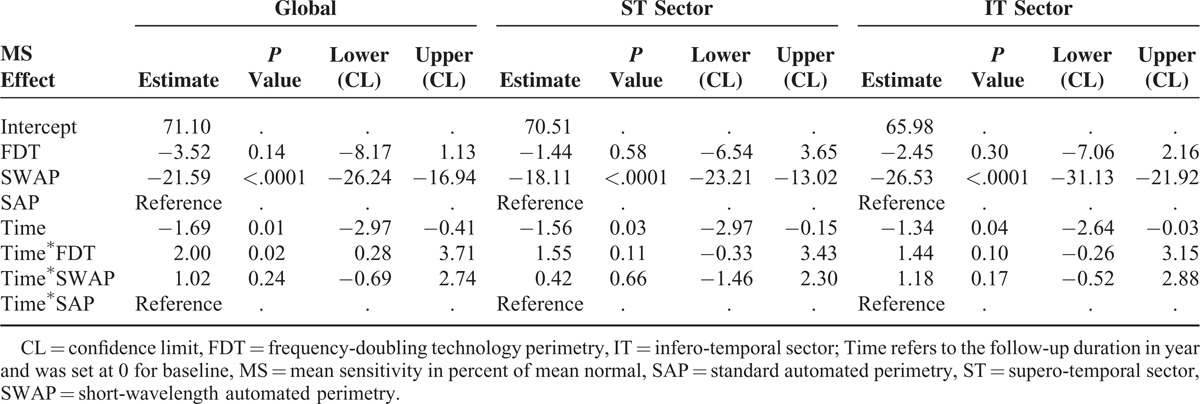
Global and Sectoral Progression Analyses Using Linear Mixed Modeling (Fixed Effects)

With SWAP, the global MS in percent of mean normal was described as, MS_SWAP-global_ (time) = 49.51 − 0.67 ^∗^ time.

A significant difference was found between baseline SAP and baseline SWAP with SWAP showing lower estimation by 21.59% of mean normal (*P* < 0.0001, 95%CL, 16.94–26.24); there was no significant difference in terms of rate of change.

With FDT, the global MS in percent of mean normal was described as, MS_FDT-global_ (time) = 67.58 +0.31 ∗ time.

There was no significant difference for the estimation of the baseline between SAP and FDT. FDT did not find a negative rate of change of global MS; the difference of 2.00% of mean normal per year compared to SAP was significant (*P* = 0.023, 95%CL, 0.28–3.71).

As shown in Table 2, the rates of change of MS in the ST and IT sectors were both significantly different from zero (*P* = 0.031, 95%CL, −2.97% to −0.15% of mean normal per year for ST sector; *P* = 0.044, 95%CL, −2.64% to −0.03% of mean normal per year for IT sector) with SAP. There was no statistically significant difference in the estimation of the rate of change of MS for these sectors between different test types. Compared to SAP, SWAP showed lower estimation of baseline MS by 18.11% of normal in the ST sector (*P* < 0.0001, 95%CL, 13.02–23.21) and 26.53% of normal in the IT sector (*P* < 0.0001, 95%CL, 21.92–31.13); FDT showed comparable estimation of baseline MS in these sectors.

## DISCUSSION

Accurate assessment of progression is essential to determine the need to modify treatment strategies and also to evaluate the visual prognosis in glaucoma eyes. There is currently no reference standard for glaucoma progression. In the present study, we did not use structural measurements as the reference to determine progression because the agreement between structure and function is poor.^30,60,61^ Progression is not always detected simultaneously by structural and functional measurements.^60,62^ Furthermore, the agreement between different structural measurements has also been shown to be poor.^61^ For PLR analyses, we used sensitivity loss ≥5% of mean normal per year for nonedge locations, and ≥10% of mean normal per year for edge locations at a significance level of *P* < 0.05 as the pointwise criteria for visual field progression. These levels were chosen to approximate the commonly accepted criteria of more than 1 dB loss per year at a significant level on nonedge locations for SAP^32,43–45,47–51^ and more than 2 dB loss per year on edge locations.^32,43,44,47^ Although these criteria may be arbitrary, these rates of progression would be enough to raise concern about the need for more aggressive treatment for an average eye. For example, visual function would be subject to complete loss in 10 years for a 50% of mean normal sensitivity location if persistently progressing at 5% of mean normal per year. Finally, we required evidence of progression at more than 1 location (and also a cluster for the conservative criterion) in order to achieve higher specificity.^51^

SAP and FDT showed comparable estimation of baseline MS. Surprisingly, SWAP showed a significantly lower estimation of baseline global MS by around 20% of mean normal than SAP and FDT although we used the same age-matched normative dataset for these tests. This difference was also confirmed in the ST and IT sectors which closely relate with the optic disc sectors that are most susceptible to glaucoma.^53,54^ This suggests that defects on SWAP were overall deeper at baseline compared to SAP and FDT. This may also be due, however, to an artifact related to the greater absorption of blue light by cataractous lens in elderly people and glaucoma eyes are more affected by cataracts. A limitation of the present study is that we cannot tell if the baseline level of SWAP is true regarding glaucomatous damage or whether they are a “false-positive” estimation of sensitivity loss due to the cataractous artifact. Although deeper baseline defects with SWAP, if true (related to glaucomatous damage), may have affected its ability to detect progression compared to SAP and FDT, the SWAP defects were not deep enough to prevent further loss to be detected.^63^ In other words, progression, if present, could still be detected with SWAP. Although SWAP may detect progression in some eyes that SAP and FDT failed to detect (eg, Figure 5, Case 2), our results did not show clear advantages with SWAP in monitoring glaucoma progression (Figures 2 and 4). Hence, the application of the current generation of SWAP-SITA to follow glaucoma patients overtime might be limited.

Consensus has not been reached about the usefulness of the Matrix FDT in detecting glaucoma progression. Meira-Freitas et al^16^ showed that the rate of FDT PSD change was predictive of development of SAP visual field loss in a cohort of glaucoma suspects, while rates of SAP PSD change were not significant predictors of FDT progression during follow-up. Based on PLR analyses, Liu et al^32^ showed that FDT detected more progressing locations than SAP and rates of FDT sensitivity change were faster than that of SAP in a cohort of glaucoma patients. They also found faster rates of FDT PSD change in glaucoma suspect and ocular hypertensive eyes compared to SAP.^33^ These studies, however, compared the tests directly, without consideration for the differences in scales. Our results are consistent with those reported by Redmond et al,^31^ who did not find evidence that FDT is more sensitive than SAP using permutation of PLR. Their method is individualized and, though different from the approach we have used in this study, is also independent of the scale used to express the visual field results. In the present study, FDT did not detect more eyes as progressing compared to SAP (Figure 2); with the linear mixed modeling, FDT failed to report a global progressing trend while SAP did.

In FDT, measurement variability does not increase in areas of reduced sensitivity.^26–28^ This feature should theoretically make FDT better at detecting progression compared to SAP. Nevertheless, the Matrix FDT has fewer discrete levels (only 15 levels, while the step size of SAP is 1 dB) than SAP, and this may affect its sensitivity in detecting glaucoma progression with trend analysis such as linear regression. An underlying assumption of linear regression is that there is a trend of gradual, linear deterioration of sensitivity in glaucoma progression. FDT, with its larger steps, may show less gradual changes compared to SAP. An early study by Haymes et al^24^ showed that the 1st generation of FDT outperformed SAP using glaucoma change probability analysis (event analysis), while the opposite occurred using linear regression. Xin et al^30^ also showed that the Matrix FDT detected more progressing eyes than SAP using event analysis (defined as changes in MD exceeding measurement variability). FDT may therefore be better suited to assess progression through event analysis rather than trend analysis.

In this study, we have shown that SAP, SWAP, and FDT detect progression in different glaucoma eyes. As shown in Figure 3, only a small portion of eyes was flagged as progressing by all 3 test types with each of the criteria; for the same eye, these tests showed disagreement in the exact test location at which progression occurred during the follow-up period (Figure 5). Further studies are needed to investigate the characteristics of the eyes classified as progressing by each test type. For example, a number of factors (eg, disease severity or the structural integrity of the optic nerve and retinal nerve fiber layer) may provide information about which visual field test to use. Our finding is also similar to those reported in previous studies for the detection of glaucoma. Indeed, it has been shown that glaucomatous visual field loss can be initially detected by different test types in different patients; in other words, different test types may identify different subsets of glaucoma patients at an early stage.^17,20^ It is unclear whether a certain subset of glaucoma patients is consistently more sensitive to one of these test types. Further studies should investigate whether a given perimetric test type performs better in monitoring progression in patients that were first detected by that same test type. If this speculation was to be verified in future studies, glaucoma suspects could be assessed with different test types when they first present to clinic, and then followed longitudinally for progression using the test with which their visual field loss was initially detected. In this way, we could use SAP, SWAP, and FDT in a selective manner.

We conducted PLR analyses in a cohort consisting of 5 to 7 visits (data points) with an average interval of 12 months between consecutive visits. More frequent visual field testing may improve the estimates of rates of sensitivity change.^32,50,61^ It is not always possible, however, to obtain frequent follow-up visits due to either limited time or financial resources in clinical practice. As for the number of data points included in our PLR, Gardiner et al^64^ have shown that using shorter series length (between 6 and 9 tests) instead of longer series may be better to monitor progression because the rate of change may vary overtime. In any case, in this study, the follow-up duration and testing intervals were the same for SAP, SWAP, and FDT and all tests were affected similarly by these factors. Another limitation of this study is that we did not assess specificity, as this was beyond the scope of our study; our goal was not to determine the sensitivity and specificity of each test with each criterion for progression, but rather to compare the 3 tests once they were expressed in comparable units. Although performing our analyses in a sample of healthy eyes would be ideal, we did not have a large enough sample of control eyes with longitudinal follow-up available to assess the specificity of our criteria.

In the present study, no statistically significant difference was observed between SAP, SWAP, and FDT using the conservative criterion with PLR analysis. Nevertheless, SAP reported less improving eyes than SWAP and FDT using the moderate criterion, and FDT detected less progressing eyes than SAP and SWAP using the liberal criterion. The agreement of progressing detection between these test types was poor. A statistically significant progressing trend of MS was observed with SAP using linear mixed modeling. Compared to SAP, there was no statistically significant difference in the rate of change with SWAP, while FDT did not detect a progressing trend. SWAP showed a significantly lower estimate of baseline MS compared to SAP and FDT. In conclusion, no evidence was found that SWAP and FDT had significant benefits over SAP in monitoring glaucoma progression. For an individual patient, glaucomatous progression might be detected by a certain type of these perimetric tests.

## References

[1] McKean-Cowdin R Wang Y Wu J Impact of visual field loss on health-related quality of life in glaucoma: the Los Angeles Latino Eye Study. *Ophthalmology* 2008; 115:941–948.e1. 17997485 10.1016/j.ophtha.2007.08.037 PMC4864605

[2] van Gestel A Webers CA Beckers HJ The relationship between visual field loss in glaucoma and health-related quality-of-life. *Eye (Lond)* 2010; 24:1759–1769. 21057519 10.1038/eye.2010.133

[3] Dacey DM Packer OS Colour coding in the primate retina: diverse cell types and cone-specific circuitry. *Curr Opin Neurobiol* 2003; 13:421–427. 12965288 10.1016/s0959-4388(03)00103-x

[4] Maddess T Hemmi JM James AC Evidence for spatial aliasing effects in the Y-like cells of the magnocellular visual pathway. *Vision Res* 1998; 38:1843–1859. 9797962 10.1016/s0042-6989(97)00344-1

[5] Swanson WH Sun H Lee BB Responses of primate retinal ganglion cells to perimetric stimuli. *Invest Ophthalmol Vis Sci* 2011; 52:764–771. 20881286 10.1167/iovs.10-6158 PMC3053105

[6] White AJ Sun H Swanson WH An examination of physiological mechanisms underlying the frequency-doubling illusion. *Invest Ophthalmol Vis Sci* 2002; 43:3590–3599. 12407172

[7] Zeppieri M Demirel S Kent K Perceived spatial frequency of sinusoidal gratings. *Optom Vis Sci* 2008; 85:318–329. 18451736 10.1097/OPX.0b013e31816be9fd

[8] Sample PA Weinreb RN Progressive color visual field loss in glaucoma. *Invest Ophthalmol Vis Sci* 1992; 33:2068–2071. 1582812

[9] Johnson CA Adams AJ Casson EJ Blue-on-yellow perimetry can predict the development of glaucomatous visual field loss. *Arch Ophthalmol* 1993; 111:645–650. 8489447 10.1001/archopht.1993.01090050079034

[10] Sample PA Bosworth CF Blumenthal EZ Visual function-specific perimetry for indirect comparison of different ganglion cell populations in glaucoma. *Invest Ophthalmol Vis Sci* 2000; 41:1783–1790. 10845599

[11] Polo V Larrosa JM Pinilla I Predictive value of short-wavelength automated perimetry: a 3-year follow-up study. *Ophthalmology* 2002; 109:761–765. 11927437 10.1016/s0161-6420(01)01014-4

[12] Landers JA Goldberg I Graham SL Detection of early visual field loss in glaucoma using frequency-doubling perimetry and short-wavelength automated perimetry. *Arch Ophthalmol* 2003; 121:1705–1710. 14662589 10.1001/archopht.121.12.1705

[13] Medeiros FA Sample PA Weinreb RN Frequency doubling technology perimetry abnormalities as predictors of glaucomatous visual field loss. *Am J Ophthalmol* 2004; 137:863–871. 15126151 10.1016/j.ajo.2003.12.009

[14] Leeprechanon N Giangiacomo A Fontana H Frequency-doubling perimetry: comparison with standard automated perimetry to detect glaucoma. *Am J Ophthalmol* 2007; 143:263–271. 17178091 10.1016/j.ajo.2006.10.033

[15] Fan X Wu LL Ma ZZ Usefulness of frequency-doubling technology for perimetrically normal eyes of open-angle glaucoma patients with unilateral field loss. *Ophthalmology* 2010; 117:1530–1537. 20466428 10.1016/j.ophtha.2009.12.034

[16] Meira-Freitas D Tatham AJ Lisboa R Predicting progression of glaucoma from rates of frequency doubling technology perimetry change. *Ophthalmology* 2014; 121:498–507. 24289917 10.1016/j.ophtha.2013.09.016 PMC3946572

[17] Sample PA Medeiros FA Racette L Identifying glaucomatous vision loss with visual-function-specific perimetry in the diagnostic innovations in glaucoma study. *Invest Ophthalmol Vis Sci* 2006; 47:3381–3389. 16877406 10.1167/iovs.05-1546

[18] Patel A Wollstein G Ishikawa H Comparison of visual field defects using matrix perimetry and standard achromatic perimetry. *Ophthalmology* 2007; 114:480–487. 17123623 10.1016/j.ophtha.2006.08.009 PMC1945823

[19] van der Schoot J Reus NJ Colen TP The ability of short-wavelength automated perimetry to predict conversion to glaucoma. *Ophthalmology* 2010; 117:30–34. 19896194 10.1016/j.ophtha.2009.06.046

[20] Liu S Lam S Weinreb RN Comparison of standard automated perimetry, frequency-doubling technology perimetry, and short-wavelength automated perimetry for detection of glaucoma. *Invest Ophthalmol Vis Sci* 2011; 52:7325–7331. 21810975 10.1167/iovs.11-7795

[21] Johnson CA Adams AJ Casson EJ Progression of early glaucomatous visual field loss as detected by blue-on-yellow and standard white-on-white automated perimetry. *Arch Ophthalmol* 1993; 111:651–656. 8489448 10.1001/archopht.1993.01090050085035

[22] Bayer AU Erb C Short wavelength automated perimetry, frequency doubling technology perimetry, and pattern electroretinography for prediction of progressive glaucomatous standard visual field defects. *Ophthalmology* 2002; 109:1009–1017. 11986111 10.1016/s0161-6420(02)01015-1

[23] Girkin CA Emdadi A Sample PA Short-wavelength automated perimetry and standard perimetry in the detection of progressive optic disc cupping. *Arch Ophthalmol* 2000; 118:1231–1236. 10980768 10.1001/archopht.118.9.1231

[24] Haymes SA Hutchison DM McCormick TA Glaucomatous visual field progression with frequency-doubling technology and standard automated perimetry in a longitudinal prospective study. *Invest Ophthalmol Vis Sci* 2005; 46:547–554. 15671281 10.1167/iovs.04-0973

[25] Bengtsson B A new rapid threshold algorithm for short-wavelength automated perimetry. *Invest Ophthalmol Vis Sci* 2003; 44:1388–1394. 12601072 10.1167/iovs.02-0169

[26] Chauhan BC Johnson CA Test-retest variability of frequency-doubling perimetry and conventional perimetry in glaucoma patients and normal subjects. *Invest Ophthalmol Vis Sci* 1999; 40:648–656. 10067968

[27] Artes PH Hutchison DM Nicolela MT Threshold and variability properties of matrix frequency-doubling technology and standard automated perimetry in glaucoma. *Invest Ophthalmol Vis Sci* 2005; 46:2451–2457. 15980235 10.1167/iovs.05-0135

[28] Wall M Woodward KR Doyle CK Repeatability of automated perimetry: a comparison between standard automated perimetry with stimulus size III and V, matrix, and motion perimetry. *Invest Ophthalmol Vis Sci* 2009; 50:974–979. 18952921 10.1167/iovs.08-1789

[29] Spry PG Johnson CA McKendrick AM Variability components of standard automated perimetry and frequency-doubling technology perimetry. *Invest Ophthalmol Vis Sci* 2001; 42:1404–1410. 11328758

[30] Xin D Greenstein VC Ritch R A comparison of functional and structural measures for identifying progression of glaucoma. *Invest Ophthalmol Vis Sci* 2011; 52:519–526. 20847115 10.1167/iovs.10-5174 PMC3053295

[31] Redmond T O’Leary N Hutchison DM Visual field progression with frequency-doubling matrix perimetry and standard automated perimetry in patients with glaucoma and in healthy controls. *JAMA Ophthalmol* 2013; 131:1565–1572. 24177807 10.1001/jamaophthalmol.2013.4382

[32] Liu S Yu M Weinreb RN Frequency doubling technology perimetry for detection of visual field progression in glaucoma: a pointwise linear regression analysis. *Invest Ophthalmol Vis Sci* 2014; 55:2862–2869. 24595388 10.1167/iovs.13-13225

[33] Liu S Yu M Weinreb RN Frequency-doubling technology perimetry for detection of the development of visual field defects in glaucoma suspect eyes: a prospective study. *JAMA Ophthalmol* 2014; 132:77–83. 24177945 10.1001/jamaophthalmol.2013.5511

[34] Sun H Dul MW Swanson WH Linearity can account for the similarity among conventional, frequency-doubling, and gabor-based perimetric tests in the glaucomatous macula. *Optom Vis Sci* 2006; 83:455–465. 16840860 10.1097/01.opx.0000225103.18087.5d PMC1752204

[35] Hot A Dul MW Swanson WH Development and evaluation of a contrast sensitivity perimetry test for patients with glaucoma. *Invest Ophthalmol Vis Sci* 2008; 49:3049–3057. 18378580 10.1167/iovs.07-1205 PMC2532064

[36] Shafi A Swanson WH Dul MW Structure and function in patients with glaucomatous defects near fixation. *Optom Vis Sci* 2011; 88:130–139. 20935585 10.1097/OPX.0b013e3181fa38f4 PMC3014396

[37] Hu R Marin-Franch I Racette L Prediction accuracy of a novel dynamic structure-function model for glaucoma progression. *Invest Ophthalmol Vis Sci* 2014; 55:8086–8094. 25358735 10.1167/iovs.14-14928 PMC4266083

[38] Sample PA Girkin CA Zangwill LM The African Descent and Glaucoma Evaluation Study (ADAGES): design and baseline data. *Arch Ophthalmol* 2009; 127:1136–1145. 19752422 10.1001/archophthalmol.2009.187 PMC2761830

[39] Racette L Liebmann JM Girkin CA African Descent and Glaucoma Evaluation Study (ADAGES): III. Ancestry differences in visual function in healthy eyes. *Arch Ophthalmol* 2010; 128:551–559. 20457975 10.1001/archophthalmol.2010.58 PMC2907156

[40] Anderson DR Patella VM Automated Static Perimetry, 2nd ed. 1999; St. Louis, Missouri: Mosby, 152–153.

[41] Peli E In search of a contrast metric: matching the perceived contrast of Gabor patches at different phases and bandwidths. *Vision Res* 1997; 37:3217–3224. 9425539 10.1016/s0042-6989(96)00262-3

[42] Fitzke FW Hitchings RA Poinoosawmy D Analysis of visual field progression in glaucoma. *Br J Ophthalmol* 1996; 80:40–48. 8664231 10.1136/bjo.80.1.40 PMC505382

[43] McNaught AI Crabb DP Fitzke FW Visual field progression: comparison of Humphrey Statpac2 and pointwise linear regression analysis. *Graefes Arch Clin Exp Ophthalmol* 1996; 234:411–418. 8817283 10.1007/BF02539406

[44] Viswanathan AC Fitzke FW Hitchings RA Early detection of visual field progression in glaucoma: a comparison of PROGRESSOR and STATPAC 2. *Br J Ophthalmol* 1997; 81:1037–1042. 9497460 10.1136/bjo.81.12.1037 PMC1722087

[45] Wilkins MR Fitzke FW Khaw PT Pointwise linear progression criteria and the detection of visual field change in a glaucoma trial. *Eye (Lond)* 2006; 20:98–106. 15650759 10.1038/sj.eye.6701781

[46] Kummet CM Zamba KD Doyle CK Refinement of pointwise linear regression criteria for determining glaucoma progression. *Invest Ophthalmol Vis Sci* 2013; 54:6234–6241. 23908183 10.1167/iovs.13-11680 PMC3778872

[47] De Moraes CG Liebmann CA Susanna R Jr Examination of the performance of different pointwise linear regression progression criteria to detect glaucomatous visual field change. *Clin Experiment Ophthalmol* 2012; 40:e190–e196. 21902781 10.1111/j.1442-9071.2011.02680.x

[48] Nouri-Mahdavi K Caprioli J Coleman AL Pointwise linear regression for evaluation of visual field outcomes and comparison with the advanced glaucoma intervention study methods. *Arch Ophthalmol* 2005; 123:193–199. 15710815 10.1001/archopht.123.2.193

[49] Nouri-Mahdavi K Hoffman D Ralli M Comparison of methods to predict visual field progression in glaucoma. *Arch Ophthalmol* 2007; 125:1176–1181. 17846355 10.1001/archopht.125.9.1176

[50] Nouri-Mahdavi K Zarei R Caprioli J Influence of visual field testing frequency on detection of glaucoma progression with trend analyses. *Arch Ophthalmol* 2011; 129:1521–1527. 21825177 10.1001/archophthalmol.2011.224

[51] Vesti E Johnson CA Chauhan BC Comparison of different methods for detecting glaucomatous visual field progression. *Invest Ophthalmol Vis Sci* 2003; 44:3873–3879. 12939303 10.1167/iovs.02-1171

[52] Heijl A Lindgren G Olsson J Normal variability of static perimetric threshold values across the central visual field. *Arch Ophthalmol* 1987; 105:1544–1549. 3675288 10.1001/archopht.1987.01060110090039

[53] Garway-Heath DF Holder GE Fitzke FW Relationship between electrophysiological, psychophysical, and anatomical measurements in glaucoma. *Invest Ophthalmol Vis Sci* 2002; 43:2213–2220. 12091419

[54] Garway-Heath DF Poinoosawmy D Fitzke FW Mapping the visual field to the optic disc in normal tension glaucoma eyes. *Ophthalmology* 2000; 107:1809–1815. 11013178 10.1016/s0161-6420(00)00284-0

[55] Hood DC Anderson SC Wall M Structure versus function in glaucoma: an application of a linear model. *Invest Ophthalmol Vis Sci* 2007; 48:3662–3668. 17652736 10.1167/iovs.06-1401

[56] Fleiss JL Statistical Methods for Rates and Proportions. 2nd ed 1981; New York: John Wiley, 38–46.

[57] Landis JR Koch GG The measurement of observer agreement for categorical data. *Biometrics* 1977; 33:159–174. 843571

[58] R Core Team. R: A Language and Environment for Statistical Computing. Vienna, Austria: R Foundation for Statistical Computing; 2014.

[59] Marín-Franch I Swanson WH The visualFields package: a tool for analysis and visualization of visual fields. *J Vis* 2013; 13:10:1–12. 10.1167/13.4.10 PMC3600987 23492926

[60] Malik R Swanson WH Garway-Heath DF ‘Structure-function relationship’ in glaucoma: past thinking and current concepts. *Clin Experiment Ophthalmol* 2012; 40:369–380. 22339936 10.1111/j.1442-9071.2012.02770.x PMC3693944

[61] Leung CK Liu S Weinreb RN Evaluation of retinal nerve fiber layer progression in glaucoma a prospective analysis with neuroretinal rim and visual field progression. *Ophthalmology* 2011; 118:1551–1557. 21529958 10.1016/j.ophtha.2010.12.035

[62] Chauhan BC Nicolela MT Artes PH Incidence and rates of visual field progression after longitudinally measured optic disc change in glaucoma. *Ophthalmology* 2009; 116:2110–2118. 19500850 10.1016/j.ophtha.2009.04.031

[63] Gardiner SK Swanson WH Goren D Assessment of the reliability of standard automated perimetry in regions of glaucomatous damage. *Ophthalmology* 2014; 121:1359–1369. 24629617 10.1016/j.ophtha.2014.01.020 PMC4082764

[64] Gardiner SK Demirel S De Moraes CG Series length used during trend analysis affects sensitivity to changes in progression rate in the ocular hypertension treatment study. *Invest Ophthalmol Vis Sci* 2013; 54:1252–1259. 23349433 10.1167/iovs.12-10218 PMC3597197

